# (1*S*,2*R*,4*S*)-1-[(Benzyl­amino)­meth­yl]-4-(prop-1-en-2-yl)cyclo­hexane-1,2-diol

**DOI:** 10.1107/S1600536810052323

**Published:** 2010-12-18

**Authors:** Rachid Outouch, Brahim Boualy, Mustapha Ait Ali, Larbi El Firdoussi, Corrado Rizzoli

**Affiliations:** a Equipe de Chimie de Coordination et Catalyse, Département de Chimie, Faculté des Sciences Semlalia, BP 2390, 40001 Marrakech, Morocco; b Dipartimento di Chimica Generale ed Inorganica, Chimica Analitica, Chimica Fisica, Universitá degli Studi di Parma, Viale G. P. Usberti 17/A, I-43100 Parma, Italy

## Abstract

The title compound, C_17_H_25_NO_2_, was synthesized by epoxidation of the double bond of (*S*)-perillyl alcohol [(*S*)-4-isopropenyl-1-cyclo­hexenyl­methanol], followed by the oxirane ring-opening by benzyl­amine using [Ca(CF_3_CO_2_)_2_] as catalyst under solvent-free condition at 313 K. The mol­ecular conformation is stabilized by an intra­molecular O—H⋯N hydrogen bond. In the crystal, mol­ecules are linked by inter­molecular N—H⋯O hydrogen bonds, forming chains parallel to the *a* axis, which are further connected by O—H⋯O hydrogen bonds into sheets parallel to (010). The absolute configuration of the mol­ecule is known from the synthetic procedure.

## Related literature

For the biological activity and applications of amino­diols, see: Alexander & Liotta (1996 ▶); Allepuz *et al.* (2010 ▶); Beaulieu *et al.* (1999 ▶); Braga *et al.* (2003 ▶); Chen *et al.* (1996 ▶); Cherng *et al.* (1995 ▶, 1999 ▶); Gondela & Walczak (2010 ▶); Kempf *et al.* (1992 ▶); Panev *et al.* (2001 ▶); Pastó *et al.* (1996 ▶); Wang *et al.* (1995 ▶). For the synthesis of amidiol derivatives, see: Ager *et al.* (1996 ▶); Bergmeier (2000 ▶); Canas *et al.* (1991 ▶); Carree *et al.* (2004 ▶); Dias *et al.* (2008 ▶); Fan & Hou (2003 ▶); Kamal *et al.* (2005 ▶); Kwon & Ko (2003 ▶); Lee & Kang (2004 ▶); Szakonyi *et al.* (2008 ▶); Zhao *et al.* (2004 ▶). For the use of [Ca(CF_3_CO_2_)_2_] as catalyst, see: Harrad *et al.* (2010 ▶). For the synthesis of (*S*)-1,2-ep­oxy­perillyl alcohol, see: Bach *et al.* (1979 ▶). For the graph-set analysis of hydrogen bonding, see: Bernstein *et al.* (1995 ▶). For details of ring-puckering analysis, see: Cremer & Pople (1975 ▶).
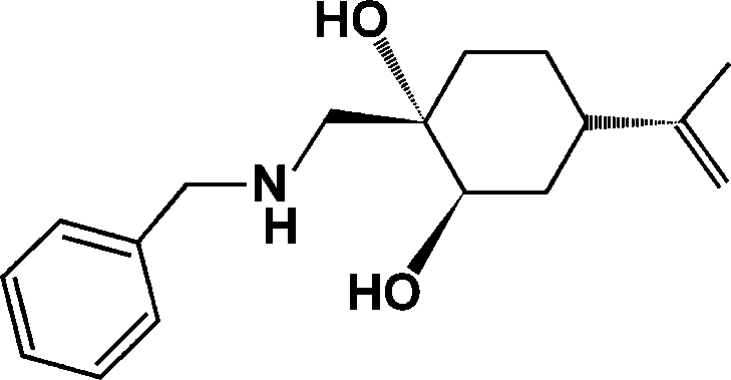


## Experimental

### 

#### Crystal data

C_17_H_25_NO_2_
*M* *_r_* = 275.38
Monoclinic, 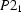

*a* = 5.8281 (4) Å
*b* = 24.5421 (16) Å
*c* = 5.8776 (4) Å
β = 105.908 (4)°
*V* = 808.50 (10) Å^3^
*Z* = 2
Cu *K*α radiation
μ = 0.58 mm^−1^
*T* = 294 K
0.20 × 0.17 × 0.15 mm

#### Data collection

Siemens AED diffractometer
3041 measured reflections
1567 independent reflections
1477 reflections with *I* > 2σ(*I*)
*R*_int_ = 0.010
3 standard reflections every 100 reflections  intensity decay: 0.02%

#### Refinement

*R*[*F* ^2^ > 2σ(*F* ^2^)] = 0.031
*wR*(*F* ^2^) = 0.092
*S* = 1.08
1567 reflections
195 parameters
1 restraint
H atoms treated by a mixture of independent and constrained refinement
Δρ_max_ = 0.16 e Å^−3^
Δρ_min_ = −0.09 e Å^−3^

### 

Data collection: *AED* (Belletti *et al.*, 1993 ▶); cell refinement: *AED*; data reduction: *AED*; program(s) used to solve structure: *SIR97* (Altomare *et al.*, 1999 ▶); program(s) used to refine structure: *SHELXL97* (Sheldrick, 2008 ▶); molecular graphics: *ORTEP-3 for Windows* (Farrugia, 1997 ▶) and *SCHAKAL97* (Keller, 1997 ▶); software used to prepare material for publication: *SHELXL97* and *PARST95* (Nardelli, 1995 ▶).

## Supplementary Material

Crystal structure: contains datablocks global, I. DOI: 10.1107/S1600536810052323/gk2332sup1.cif

Structure factors: contains datablocks I. DOI: 10.1107/S1600536810052323/gk2332Isup2.hkl

Additional supplementary materials:  crystallographic information; 3D view; checkCIF report

## Figures and Tables

**Table 1:** Hydrogen-bond geometry (Å, °)

*D*—H⋯*A*	*D*—H	H⋯*A*	*D*⋯*A*	*D*—H⋯*A*
O2—H2*O*⋯N1	0.90 (3)	1.88 (3)	2.676 (2)	147 (3)
O1—H1*O*⋯O2^i^	0.83 (3)	1.89 (3)	2.721 (2)	171 (3)
N1—H1*N*⋯O1^ii^	0.92 (2)	2.15 (2)	3.037 (2)	164 (2)

Symmetry codes: (i) 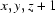; (ii) 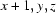.

**Fig. 1.**
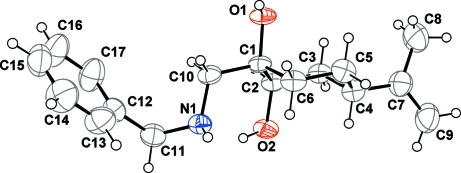
The molecular structure of the title compound, with displacement ellipsoids drawn at the 40% probability level.

**Fig. 2.**
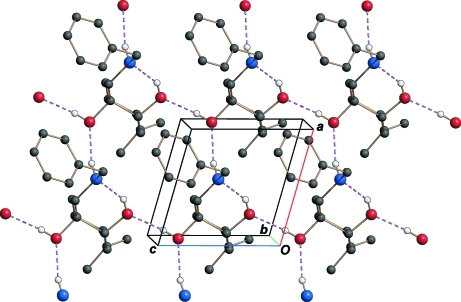
Partial crystal packing of the title compound viewed approximately along the a axis. Intra- and intermolecular hydrogen bonds are shown as dashed lines. H atoms not involved in hydrogen bondings are omitted for clarity.

## supplementary crystallographic information

### Comment

Aminodiols play important roles in drug therapy and drug research. For example, 5-(ω-hydroxyalkylamino) derivatives of mucochloric acids (2,3-dichloro-4-oxo-2-butenoic acid) have antibacterial and antiprotozoal activities (Gondela & Walczak, 2010). Other aminodiols have been found to act as HIV protease inhibitors (Kempf *et al.*, 1992; Wang *et al.*, 1995; Chen *et al.*, 1996), or to exert renin inhibitor activity (Alexander & Liotta, 1996; Beaulieu *et al.*, 1999). Furthermore, aminodiols may serve as useful starting materials for the synthesis of biologically active compounds, e. g. 3-amino-1,2-butanediol derivatives, which are key intermediates in the synthesis of the anticancer agent ES-285 (Allepuz *et al.*, 2010). Chiral aminodiols and their derivatives also find excellent applications as catalysts for enantioselective transformations (Panev *et al.*, 2001; Cherng *et al.*, 1995, 1999; Pastó *et al.*, 1996; Braga *et al.*, 2003).

Among many different approaches developed for the synthesis of aminodiol derivatives (Canas *et al.*, 1991; Panev *et al.*, 2001; Kwon & Ko, 2003; Dias *et al.*, 2008; Szakonyi *et al.*, 2008), aminolysis of 1,2-epoxides represents one of the most valuable pathway to produce commercially important aminoalcohols and aminodiols from olefins (Ager *et al.*, 1996; Bergmeier, 2000; Lee & Kang, 2004; Zhao *et al.*, 2004; Fan & Hou, 2003; Kamal *et al.*, 2005; Carree *et al.*, 2004). As a contribution to this widespread area, we describe here the synthesis and crystal structure of the title new aminodiol derivative of perillyl alcohol. The synthetic methodology involves an epoxidation of the double bond, followed by the oxirane ring-opening by using benzylamine and Ca(CF_3_CO_2_)_2_ as catalyst (Harrad *et al.*, 2010) under solvent-free condition at 40°C.

The molecular structure of the title compound is shown in Fig. 1. The cyclohexane ring is in a chair conformation, with puckering parameters *Q*, θ and φ of 0.560 (2) Å, 2.6 (2)° and -167 (4)°, respectively (Cremer & Pople, 1975). The hydroxy groups at atoms C1 and C2 are both in axial positions. The molecular conformation is stabilized by an intramolecular O—H···N hydrogen bond (Table 1), generating a ring of *S*(6) graph set motif (Bernstein *et al.*, 1995). In the crystal structure (Fig. 2), molecules are linked into chains parallel to the *a* axis by intermolecular N—H···O hydrogen bonds. The chains are further connected *via* O—H···O hydrogen bonding interactions to form sheets parallel to (010).

### Experimental

(*S*)-1,2-Epoxyperillyl alcohol was prepared from commercially available (*S*)-perillyl alcohol [(*S*)-4-isopropenyl-1-cyclohexenylmethanol, Fluka] using the procedure described in the literature (Bach *et al.*, 1979). A mixture of benzylamine (5.1 mmol) and (*S*)-1,2-epoxyperillyl alcohol (5 mmol) was added to Ca(CF_3_CO_2_)_2_ (0.25 mmol) under solvent-free conditions. The mixture was stirred at 40 °C for 72 h, then it was extracted with ethyl acetate (3 × 10 ml), dried over Na_2_SO_4_ and the solvent was removed under reduced pressure. Column chromatography (column 60 *x* 2.5 cm, hexane) of the residue on silica gel gave the title aminodiol in 52% yield (m. p. 429-430 K). Colourless crystals suitable for X-ray analysis were obtained on slow evaporation of the solvent.

### Refinement

The hydroxy and amine H atoms were located in a difference Fourier map and refined freely. All other H atoms were placed at calculated positions and refined using the riding model approximation, with C—H = 0.93–0.98 Å, and with *U*_iso_(H) = 1.2 *U*_eq_(C) or 1.5 *U*_eq_(C) for methyl H atoms. In the absence of significant anomalous scattering effects, the 1237 Friedel pairs were merged in the last cycles of refinement. The absolute configuration was assigned on the basis of the known configuration of the perillyl alcohol employed in the synthesis.

### Crystal data

**Table:**

C_17_H_25_NO_2_	*F*(000) = 300
*M**_r_* = 275.38	*D*_x_ = 1.131 Mg m^−^^3^
Monoclinic, *P*2_1_	Cu *K*α radiation, λ = 1.54178 Å
Hall symbol: P 2yb	Cell parameters from 48 reflections
*a* = 5.8281 (4) Å	θ = 12.6–38.8°
*b* = 24.5421 (16) Å	µ = 0.58 mm^−^^1^
*c* = 5.8776 (4) Å	*T* = 294 K
β = 105.908 (4)°	Block, colourless
*V* = 808.50 (10) Å^3^	0.20 × 0.17 × 0.15 mm
*Z* = 2	

### Data collection

**Table:**

Siemens AED diffractometer	*R*_int_ = 0.010
Radiation source: fine-focus sealed tube	θ_max_ = 69.8°, θ_min_ = 3.6°
graphite	*h* = −7→7
θ/2θ scans	*k* = −28→29
3041 measured reflections	*l* = −7→1
1567 independent reflections	3 standard reflections every 100 reflections
1477 reflections with *I* > 2σ(*I*)	intensity decay: 0.02%

### Refinement

**Table:**

Refinement on *F*^2^	Secondary atom site location: difference Fourier map
Least-squares matrix: full	Hydrogen site location: inferred from neighbouring sites
*R*[*F*^2^ > 2σ(*F*^2^)] = 0.031	H atoms treated by a mixture of independent and constrained refinement
*wR*(*F*^2^) = 0.092	*w* = 1/[σ^2^(*F*_o_^2^) + (0.0584*P*)^2^ + 0.0328*P*] where *P* = (*F*_o_^2^ + 2*F*_c_^2^)/3
*S* = 1.08	(Δ/σ)_max_ < 0.001
1567 reflections	Δρ_max_ = 0.16 e Å^−^^3^
195 parameters	Δρ_min_ = −0.09 e Å^−^^3^
1 restraint	Extinction correction: *SHELXL97* (Sheldrick, 2008)
Primary atom site location: structure-invariant direct methods	Extinction coefficient: 0.0091 (17)

### Special details

**Table:**

Refinement. Refinement of *F*^2^ against ALL reflections. The weighted *R*-factor *wR* and goodness of fit *S* are based on *F*^2^, conventional *R*-factors *R* are based on *F*, with *F* set to zero for negative *F*^2^. The threshold expression of *F*^2^ > σ(*F*^2^) is used only for calculating *R*-factors(gt) *etc*. and is not relevant to the choice of reflections for refinement. *R*-factors based on *F*^2^ are statistically about twice as large as those based on *F*, and *R*- factors based on ALL data will be even larger.

### Fractional atomic coordinates and isotropic or equivalent isotropic displacement parameters (Å^2^)

**Table:**

	*x*	*y*	*z*	*U*_iso_*/*U*_eq_	
O1	0.0402 (2)	0.20566 (7)	0.8285 (2)	0.0523 (3)	
H1O	0.114 (5)	0.2089 (13)	0.971 (5)	0.075 (7)*	
O2	0.2514 (2)	0.20737 (7)	0.3026 (2)	0.0573 (4)	
H2O	0.379 (6)	0.1870 (13)	0.371 (6)	0.090 (9)*	
N1	0.5565 (3)	0.15127 (7)	0.6459 (3)	0.0519 (4)	
H1N	0.688 (4)	0.1715 (10)	0.719 (4)	0.055 (6)*	
C1	0.2235 (3)	0.20879 (8)	0.7095 (2)	0.0432 (4)	
C2	0.0876 (3)	0.21090 (9)	0.4445 (3)	0.0451 (4)	
H2	−0.0219	0.1798	0.4075	0.054*	
C3	−0.0551 (3)	0.26297 (9)	0.3798 (3)	0.0501 (4)	
H3A	−0.1818	0.2634	0.4578	0.060*	
H3B	−0.1284	0.2636	0.2104	0.060*	
C4	0.0993 (4)	0.31396 (9)	0.4509 (4)	0.0551 (5)	
H4	0.2232	0.3127	0.3669	0.066*	
C5	0.2265 (4)	0.31182 (9)	0.7172 (4)	0.0580 (5)	
H5A	0.3308	0.3432	0.7602	0.070*	
H5B	0.1087	0.3137	0.8059	0.070*	
C6	0.3724 (3)	0.25995 (9)	0.7830 (3)	0.0509 (4)	
H6A	0.5009	0.2602	0.7073	0.061*	
H6B	0.4433	0.2593	0.9527	0.061*	
C7	−0.0400 (5)	0.36592 (11)	0.3773 (5)	0.0715 (6)	
C8	−0.2383 (6)	0.37780 (15)	0.4831 (8)	0.1137 (13)	
H8A	−0.3043	0.4130	0.4311	0.170*	
H8B	−0.1791	0.3776	0.6524	0.170*	
H8C	−0.3599	0.3505	0.4342	0.170*	
C9	0.0179 (7)	0.40014 (13)	0.2250 (6)	0.0967 (10)	
H9A	−0.0669	0.4324	0.1828	0.116*	
H9B	0.1431	0.3917	0.1613	0.116*	
C10	0.3697 (3)	0.15611 (9)	0.7684 (3)	0.0519 (5)	
H10A	0.4431	0.1547	0.9377	0.062*	
H10B	0.2628	0.1252	0.7264	0.062*	
C11	0.6236 (4)	0.09492 (10)	0.6142 (4)	0.0654 (6)	
H11A	0.7594	0.0955	0.5487	0.078*	
H11B	0.4925	0.0778	0.4979	0.078*	
C12	0.6868 (4)	0.05945 (9)	0.8337 (4)	0.0606 (5)	
C13	0.9001 (5)	0.06545 (13)	1.0055 (6)	0.0835 (8)	
H13	1.0082	0.0918	0.9871	0.100*	
C14	0.9548 (6)	0.03319 (17)	1.2024 (6)	0.1002 (10)	
H14	1.1006	0.0377	1.3152	0.120*	
C15	0.8001 (7)	−0.00523 (12)	1.2363 (6)	0.0908 (9)	
H15	0.8403	−0.0274	1.3698	0.109*	
C16	0.5866 (7)	−0.01100 (13)	1.0734 (7)	0.1009 (10)	
H16	0.4775	−0.0366	1.0966	0.121*	
C17	0.5310 (6)	0.02089 (12)	0.8739 (6)	0.0914 (9)	
H17	0.3842	0.0163	0.7629	0.110*	

### Atomic displacement parameters (Å^2^)

**Table:**

	*U*^11^	*U*^22^	*U*^33^	*U*^12^	*U*^13^	*U*^23^
O1	0.0435 (6)	0.0846 (9)	0.0303 (6)	−0.0047 (7)	0.0128 (5)	−0.0006 (6)
O2	0.0604 (7)	0.0859 (10)	0.0274 (5)	0.0089 (8)	0.0153 (5)	0.0031 (7)
N1	0.0461 (8)	0.0652 (10)	0.0451 (8)	0.0007 (7)	0.0137 (6)	0.0022 (7)
C1	0.0379 (7)	0.0653 (10)	0.0260 (7)	−0.0025 (8)	0.0085 (5)	0.0020 (8)
C2	0.0444 (8)	0.0633 (10)	0.0261 (7)	−0.0068 (8)	0.0070 (6)	−0.0051 (8)
C3	0.0436 (8)	0.0705 (11)	0.0320 (7)	−0.0022 (8)	0.0033 (6)	0.0000 (8)
C4	0.0514 (9)	0.0625 (11)	0.0504 (10)	−0.0026 (9)	0.0121 (8)	0.0001 (9)
C5	0.0557 (10)	0.0649 (12)	0.0496 (11)	−0.0066 (9)	0.0079 (8)	−0.0123 (9)
C6	0.0421 (8)	0.0730 (12)	0.0337 (8)	−0.0071 (9)	0.0037 (6)	−0.0057 (9)
C7	0.0732 (14)	0.0707 (14)	0.0665 (14)	0.0036 (11)	0.0122 (11)	0.0052 (11)
C8	0.089 (2)	0.092 (2)	0.169 (4)	0.0326 (18)	0.051 (2)	0.028 (2)
C9	0.134 (3)	0.0738 (17)	0.084 (2)	0.0154 (17)	0.0331 (19)	0.0160 (15)
C10	0.0495 (10)	0.0702 (12)	0.0365 (9)	0.0002 (8)	0.0126 (7)	0.0074 (8)
C11	0.0692 (13)	0.0728 (14)	0.0557 (11)	−0.0016 (10)	0.0196 (10)	−0.0077 (10)
C12	0.0617 (11)	0.0563 (11)	0.0642 (13)	0.0049 (9)	0.0179 (9)	−0.0071 (10)
C13	0.0620 (13)	0.103 (2)	0.0823 (17)	−0.0091 (13)	0.0138 (12)	0.0109 (15)
C14	0.0825 (18)	0.120 (3)	0.086 (2)	0.0014 (18)	0.0019 (15)	0.0123 (19)
C15	0.115 (2)	0.0723 (16)	0.0830 (19)	0.0220 (16)	0.0235 (17)	0.0135 (13)
C16	0.107 (2)	0.0737 (17)	0.116 (3)	−0.0139 (16)	0.021 (2)	0.0218 (17)
C17	0.0863 (17)	0.0703 (16)	0.102 (2)	−0.0157 (13)	−0.0009 (15)	0.0099 (15)

### Geometric parameters (Å, °)

**Table:**

O1—C1	1.4301 (18)	C7—C9	1.336 (4)
O1—H1O	0.83 (3)	C7—C8	1.484 (5)
O2—C2	1.4319 (19)	C8—H8A	0.9600
O2—H2O	0.89 (3)	C8—H8B	0.9600
N1—C11	1.463 (3)	C8—H8C	0.9600
N1—C10	1.465 (2)	C9—H9A	0.9300
N1—H1N	0.92 (2)	C9—H9B	0.9300
C1—C6	1.520 (3)	C10—H10A	0.9700
C1—C10	1.535 (3)	C10—H10B	0.9700
C1—C2	1.5426 (19)	C11—C12	1.516 (3)
C2—C3	1.515 (3)	C11—H11A	0.9700
C2—H2	0.9800	C11—H11B	0.9700
C3—C4	1.531 (3)	C12—C17	1.376 (4)
C3—H3A	0.9700	C12—C13	1.378 (4)
C3—H3B	0.9700	C13—C14	1.366 (5)
C4—C7	1.510 (3)	C13—H13	0.9300
C4—C5	1.538 (3)	C14—C15	1.356 (5)
C4—H4	0.9800	C14—H14	0.9300
C5—C6	1.521 (3)	C15—C16	1.354 (5)
C5—H5A	0.9700	C15—H15	0.9300
C5—H5B	0.9700	C16—C17	1.373 (5)
C6—H6A	0.9700	C16—H16	0.9300
C6—H6B	0.9700	C17—H17	0.9300
			
C1—O1—H1O	103.5 (18)	C9—C7—C4	120.5 (3)
C2—O2—H2O	112 (2)	C8—C7—C4	117.7 (2)
C11—N1—C10	113.58 (17)	C7—C8—H8A	109.5
C11—N1—H1N	110.8 (14)	C7—C8—H8B	109.5
C10—N1—H1N	111.3 (15)	H8A—C8—H8B	109.5
O1—C1—C6	110.51 (15)	C7—C8—H8C	109.5
O1—C1—C10	106.71 (15)	H8A—C8—H8C	109.5
C6—C1—C10	113.15 (13)	H8B—C8—H8C	109.5
O1—C1—C2	104.49 (11)	C7—C9—H9A	120.0
C6—C1—C2	110.73 (15)	C7—C9—H9B	120.0
C10—C1—C2	110.82 (15)	H9A—C9—H9B	120.0
O2—C2—C3	108.21 (15)	N1—C10—C1	113.52 (15)
O2—C2—C1	110.28 (12)	N1—C10—H10A	108.9
C3—C2—C1	112.11 (15)	C1—C10—H10A	108.9
O2—C2—H2	108.7	N1—C10—H10B	108.9
C3—C2—H2	108.7	C1—C10—H10B	108.9
C1—C2—H2	108.7	H10A—C10—H10B	107.7
C2—C3—C4	112.31 (14)	N1—C11—C12	116.44 (18)
C2—C3—H3A	109.1	N1—C11—H11A	108.2
C4—C3—H3A	109.1	C12—C11—H11A	108.2
C2—C3—H3B	109.1	N1—C11—H11B	108.2
C4—C3—H3B	109.1	C12—C11—H11B	108.2
H3A—C3—H3B	107.9	H11A—C11—H11B	107.3
C7—C4—C3	112.49 (17)	C17—C12—C13	116.9 (2)
C7—C4—C5	113.05 (18)	C17—C12—C11	121.6 (2)
C3—C4—C5	109.44 (16)	C13—C12—C11	121.5 (2)
C7—C4—H4	107.2	C14—C13—C12	120.9 (3)
C3—C4—H4	107.2	C14—C13—H13	119.6
C5—C4—H4	107.2	C12—C13—H13	119.6
C6—C5—C4	111.52 (17)	C15—C14—C13	121.1 (3)
C6—C5—H5A	109.3	C15—C14—H14	119.4
C4—C5—H5A	109.3	C13—C14—H14	119.4
C6—C5—H5B	109.3	C16—C15—C14	119.2 (3)
C4—C5—H5B	109.3	C16—C15—H15	120.4
H5A—C5—H5B	108.0	C14—C15—H15	120.4
C1—C6—C5	112.55 (15)	C15—C16—C17	120.0 (3)
C1—C6—H6A	109.1	C15—C16—H16	120.0
C5—C6—H6A	109.1	C17—C16—H16	120.0
C1—C6—H6B	109.1	C16—C17—C12	121.8 (3)
C5—C6—H6B	109.1	C16—C17—H17	119.1
H6A—C6—H6B	107.8	C12—C17—H17	119.1
C9—C7—C8	121.7 (3)		

### Hydrogen-bond geometry (Å, °)

**Table:**

*D*—H···*A*	*D*—H	H···*A*	*D*···*A*	*D*—H···*A*
O2—H2O···N1	0.90 (3)	1.88 (3)	2.676 (2)	147 (3)
O1—H1O···O2^i^	0.83 (3)	1.89 (3)	2.721 (2)	171 (3)
N1—H1N···O1^ii^	0.92 (2)	2.15 (2)	3.037 (2)	164 (2)

Symmetry codes: (i) *x*, *y*, *z*+1; (ii) *x*+1, *y*, *z*.
